# *Aconitum carmichaelii* Debx. Attenuates Heart Failure through Inhibiting Inflammation and Abnormal Vascular Remodeling

**DOI:** 10.3390/ijms24065838

**Published:** 2023-03-19

**Authors:** Ziwei Xing, Junren Chen, Tingting Yu, Xu Li, Wei Dong, Cheng Peng, Dan Li

**Affiliations:** State Key Laboratory of Southwestern Chinese Medicine Resources, School of Pharmacy, Chengdu University of Traditional Chinese Medicine, Chengdu 611137, China

**Keywords:** *Aconitum carmichaelii* Debx., heart failure, vascular remodeling, inflammation, NF-κB signaling pathway, FOXO1, Angprotein-2

## Abstract

Heart failure (HF) is the most common complication following myocardial infarction, closely associated with ventricular remodeling. *Aconitum carmichaelii* Debx., a traditional Chinese herb, possesses therapeutic effects on HF and related cardiac diseases. However, its effects and mechanisms on HF-associated cardiac diseases are still unclear. In the present study, a water extraction of toasted *Aconitum carmichaelii* Debx. (WETA) was verified using UPLC-Q/TOF-MS. The heart function of HF rats was assessed by echocardiography and strain analysis, and myocardial injury was measured by serum levels of CK-MB, cTnT, and cTnI. The pathological changes of cardiac tissues were evaluated by 2,3,5-triphenyltetrazolium chloride (TTC) staining, hematoxylin and eosin (H&E) staining, and Masson’s trichrome staining. Additionally, the levels of inflammation-related genes and proteins and components related to vascular remodeling were detected by RT-qPCR, Western blot, and immunofluorescence. WETA significantly inhibited the changes in echocardiographic parameters and the increase in heart weight, cardiac infarction size, the myonecrosis, edema, and infiltration of inflammatory cells, collagen deposition in heart tissues, and also mitigated the elevated serum levels of CK-MB, cTnT, and cTnI in ISO-induced rats. Additionally, WETA suppressed the expressions of inflammatory genes, including IL-1β, IL-6, and TNF-α and vascular injury-related genes, such as VCAM1, ICAM1, ANP, BNP, and MHC in heart tissues of ISO-induced HF rats, which were further confirmed by Western blotting and immunofluorescence. In summary, the myocardial protective effect of WETA was conferred through inhibiting inflammatory responses and abnormal vascular remodeling in ISO-treated rats.

## 1. Introduction

Heart failure (HF), one of the deadliest diseases globally, affects approximately 40 million people worldwide [1]. The most common risk factors of HF are cardiovascular diseases, including arrhythmia, hypertension, myocardial ischemia, and cardiac hypertrophy disorders [2], which lead to a poor quality of life [3]. Despite extraordinary advances in the diagnosis (such as chest X-rays, electrocardiograms, and echocardiograms) and therapies like cell therapy, gene therapy, and interventional therapies for HF, the morbidity and mortality of HF still remain very high [4]. Therefore, the discovery of novel drug candidates is paramount to reducing the incidence and prevalence of HF. Traditional Chinese medicine has the characteristics of good efficacy, minimal side effects, and effects on multiple targets.

Myocardial inflammation and vascular abnormal injury are known to be critical factors in the development of HF [5,6]. Inflammatory responses in heart tissue are characterized by the infiltration of immune cells (e.g., macrophages) [7] and the production of pro-inflammatory cytokines and cell adhesion molecules [8], which evokes vascular damage and the expressions of VCAM-1 and ICAM-1 [9], result in aggravating the pathological process of left ventricular (LV) remodeling, finally leading to cardiac dysfunction [10]. Furthermore, in the onset of cardiac injury, increased neovascularization is a response to cardiomyocyte hypertrophy [11,12]; however, prolonged pathological stimulation leads to excessive angiogenesis, resulting in the exacerbation of structural damage to the heart [13]. Angprotein-2 (Angpt2) is a marker of vascular instability involved in cardiac vascular overproduction [14] and participates in the upregulation of pro-inflammatory macrophage infiltration as well as adverse vascular remodeling during the LV remodeling phase [15]. Additionally, a necrotic myocardium gives rise to the increased expression of FOXO1, an upstream regulator of Angpt2, which in turn accelerates cardiomyocyte hypertrophy and perivascular fibrosis [16]. Hence, inhibition of inflammation and vascular injury in heart tissues may be promising strategies for the treatment of HF-related diseases. 

*Aconitum carmichaelii* Debx., also known as fuzi in China, has been successfully applied in clinics for centuries in China to treat rheumatoid arthritis, cardiovascular disease, tumors, and other diseases such as gastroenteritis as well as edema [17]. In recent decades, its ability to treat chronic and acute heart failure has been received increasing attention. Fuzi polysaccharide could inhibit cardiomyocyte apoptosis by suppressing oxidative stress and stabilizing the mitochondrial membrane potential of cardiomyocytes, inhibiting peroxidation, and optimizing left ventricular diastolic function [18]. In addition, a water-soluble aconite alkaloid extract improved LV function, hypertrophy, fibrosis, and apoptosis by regulating calcium signaling [19]. Moreover, Fuzi activates the PPARα/PGC-1α/Sirt3 pathway and promotes mitochondrial energy metabolism in a rat model of HF [20]. Although *Aconitum carmichaelii* Debx. possesses many therapeutic properties, it contains dibasic diterpene alkaloids, considered to be the main toxins in *Aconitum carmichaelii* Debx., which limits its clinical applications and approval [21]. Therefore, in the current study, we established an ISO-induced HF rat model to explore the therapeutic roles and potential mechanisms of *Aconitum carmichaelii* Debx. in HF.

## 2. Result

### 2.1. Analysis of the Chemical Composition of WETA 

**Figure 1:**
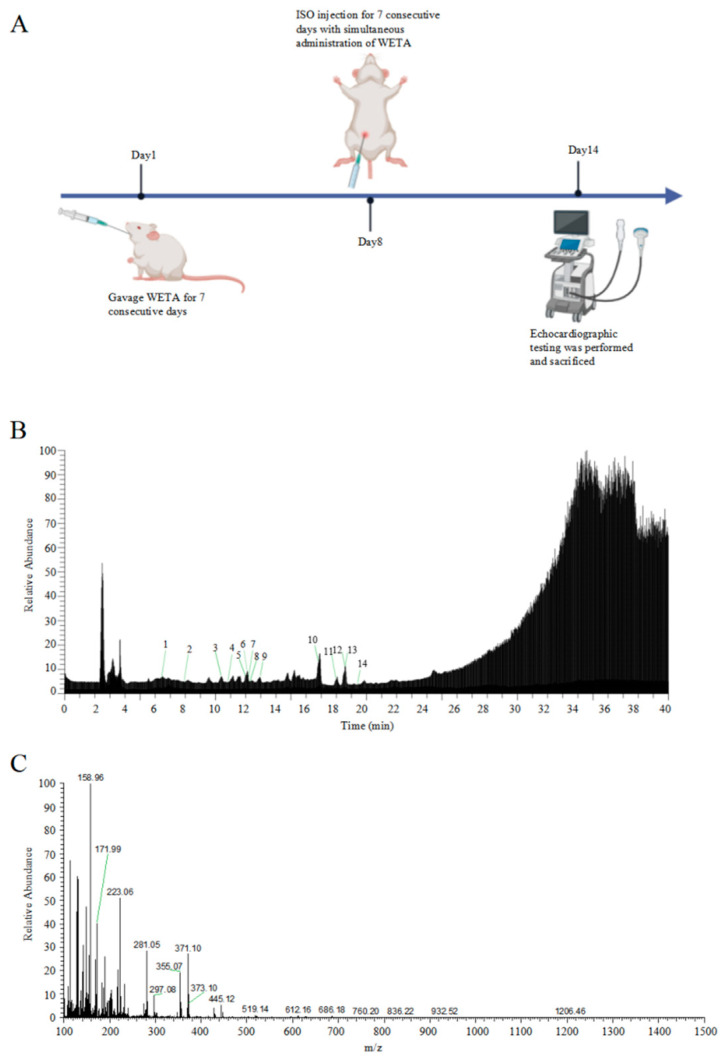
Identification of WETA compounds using LC-MS. (**A**) The experimental design flow, (**B**) total ion chromatogram of WETA, (**C**) MS^2^ spectrum of WETA. 1, Salsolinol; 2, Karakoline; 3, Isotalatizidine; 4, Napelline; 5, Neoline/Bullatine B; 6, Neoline; 7, Lycoctonine; 8, Fuziline (15-α-Hydroxyneoline); 9, Talatisamine; 10, Benzoylmesaconine; 11, Benzoylaconine; 12, Hypaconitine; 13, Benzoylhypaconine; 14, Aconitine.

The total ion chromatograms and secondary debris ion peaks are shown in Figure 1B,C. A total of 14 bioactive compounds corresponding to previous studies [22] were identified in WETA by LC ESI-MS/MS and are shown in Table 1.

### 2.2. Effects of WETA on LV Hypertrophy and Systolic Function 

**Figure 2:**
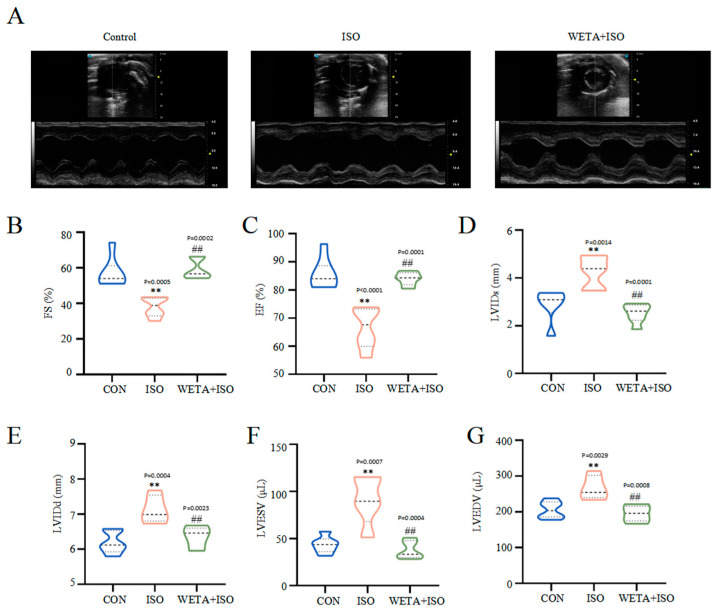
WETA improved LV echocardiography parameters in ISO-induced rats. (**A**) Representative short axis image of the LV, the levels of (**B**) FS, (**C**) EF, (**D**) LVIDs, (**E**) LVIDd, (**F**) LVESV, and (**G**) LVEDV. Values are mean ± SD (n = 6), ** *p* < 0.01 vs. CON group; ^##^ *p* < 0.01 vs. ISO group, using one-way ANOVA.

Firstly, cardiac function was detected by echocardiography. Compared with the CON group, the decreased levels of ES and FS, and increased levels of LVIDs, LVIDd, LVESV, and LVEDV in the ISO group (*p* < 0.01) indicated the impairment of LV morphology and systolic function (Figure 2A–G). Additionally, treatment with WETA enhanced LV systolic function, and decreased LV diameter and LV volume expansion (*p* < 0.01) (Figure 2A–G).

### 2.3. Effects of WETA on Segmental Myocardial Strain Caused by ISO

**Figure 3:**
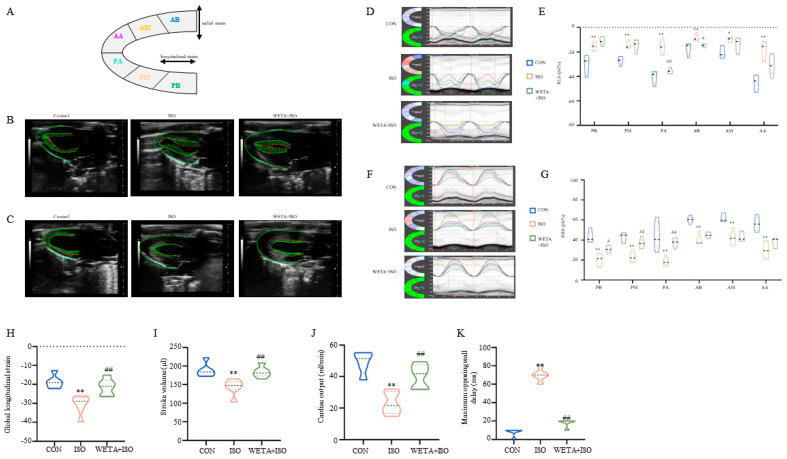
WETA relieved LV segmental myocardial strain in ISO-induced rats. (**A**) Schematic diagram of the division of the LV into six parts. Representative trace tendency of the LV wall during systole (**B**) and diastole (**C**); (**D**) representative images of RLS, (**E**) RLS of six segments. (**F**) Representative images of RRS, (**G**) RRS of six segments, (**H**) global longitudinal strain, (**I**) stroke volume, (**J**) cardiac output, (**K**) maximum opposing wall delay. AB, anterior base; AM, anterior middle; AP, anterior apex; PB, posterior base; PM, posterior middle; PA, posterior apex, RRS, regional radial strain, RLS, regional longitudinal strain. Values are mean ± SD (n = 6), * *p* < 0.05, ** *p* < 0.01 vs. CON group; ^#^ *p* < 0.05, ^##^ *p* < 0.01 vs. ISO group, using one-way ANOVA.

As shown in Figure 3A, the long axis of the LV was automatically segmented into six parts by the strain software. The typical trace tendency of the LV wall is displayed in Figure 3B,C, which exhibits the systolic and diastolic motion trajectories of global RLS and RRS, and revealed the global systolic and diastole function of the LV. Similarly, the peak time of the six segments was altered with ISO administration, which was mitigated by WETA (Figure 3D–G). According to the analysis, ISO induced a significant improvement in RLS (*p* < 0.05 or *p* < 0.01), global longitudinal strain (*p* < 0.01), and RRS (*p* < 0.01) for these six segments (Figure 3D–K). Among these, the global longitudinal strain and the PA and AB segments of RLS were significantly mitigated (*p* < 0.05, *p* < 0.01) by WETA treatment, and the PB, PM, and PA segments of RRS were increased (*p* < 0.05 or *p* < 0.01) after WETA administration (Figure 3D–K). The maximum opposing wall delay was also analyzed and found to be significantly increased in the ISO group, which was alleviated by WETA administration (Figure 3K), suggesting that WETA can improve contractile synchrony defects in the rat HF model. The above results indicated that WETA improved the movement and function of the LV in ISO rats.

### 2.4. Effects of WETA on Cardiac Morphology and Serum Heart Function Indicators

**Figure 4:**
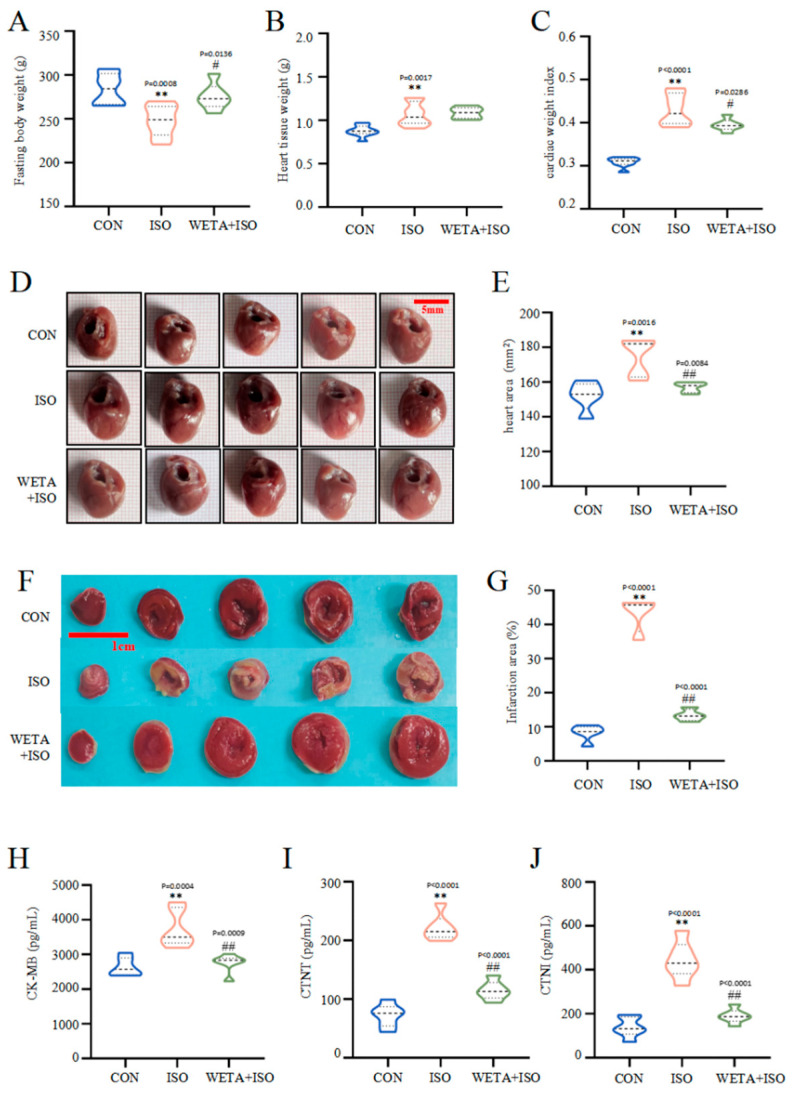
WETA reduced heart deformation and cardiac damage markers. (**A**) Fasting body weight, (**B**) heart tissue weight, (**C**) cardiac tissue index (cardiac weight index (CWI) was calculated according to the following formula: CWI(g/g) = heart tissue weight (g)/body weight (g) × 100), (**D**) representative heart shape (scale bar = 500 μm), (**E**) heart area, (**F**) representative TTC-stained sections (Scale bar = 1 cm), (**G**) heart infarction area, the serum levels of CK-MB (**H**), cTNT (**I**), and cTNI (**J**). Values are mean ± SD (n = 6), ** *p* < 0.01 vs. CON group; ^##^ *p* < 0.01, ^#^ *p* < 0.05 vs. ISO group, using one-way ANOVA.

Macroscopically, the rats in the ISO group showed reduced fasting body weight (*p* < 0.01), increased heart tissue weight (*p* < 0.01), escalated cardiac tissue index (*p* < 0.01), and enlarged heart tissues (*p* < 0.01). However, fasting body weight increased (*p* < 0.05) after WETA administration, while heart tissue weight and the cardiac tissue index (*p* < 0.05) decreased. (Figure 4A–F). TTC staining (Figure 4F) illustrated that the myocardium in the CON group was a normal red color, while the ISO group showed a large infarction area compared to the CON group (*p* < 0.01), and the infarction size was significantly reduced after WETA treatment (*p* < 0.01). 

Moreover, in rats in the ISO group, the levels of CK-MB, cTNT, and cTNI in the serum were notably higher than those in CON group (*p* < 0.01), while WETA administration caused a decrease in CK-MB, cTNT, and cTNI (*p* < 0.01) (Figure 4H–J). These evidences suggested that WETA could alleviate ISO-induced cardiac morphological changes and cardiac damage.

### 2.5. Effects of WETA on Attenuating Histopathological Changes

**Figure 5:**
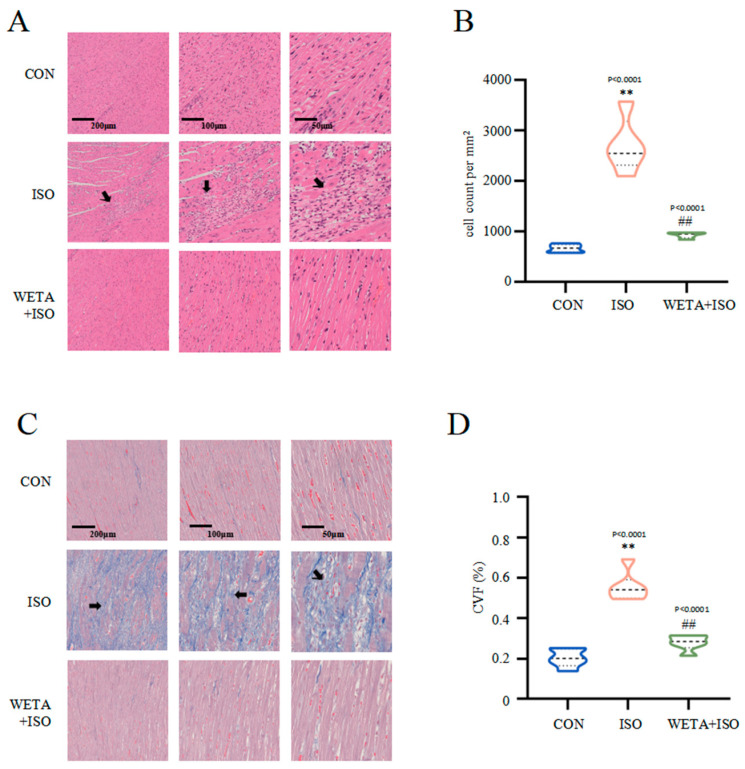
WETA attenuates histopathological changes. (**A**) H&E staining images of LV (From left to right, scale bar = 200 μm, 100 μm, 50 μm), (**B**) Positive cells in the H&E stained slices were normalized and counted as cells/mm^2^, (**C**) Masson’s staining images of LV (From left to right, scale bar = 200 μm, 100 μm, 50 μm), and (**D**) CVF of LV. Scale bar = 200 μm, 200 μm, 50 μm. Black arrows: irregular cross striations of the myocardial tissue. Values are mean ± SD (n = 6), ** *p* < 0.01 vs. CON group; ^##^ *p* < 0.01 vs. ISO group, from One-way ANOVA.

Microscope images with different magnifications for the experimental groups are shown in Figure 5. H&E staining (Figure 5A,B) showed that the cross striations of the myocardial tissue were arranged in an orderly manner and cardiomyocyte size was uniform in the CON group. In contrast, ISO treatment caused increased cardiomyocyte area, partial myocardial necrosis, severe inflammatory infiltration, and apoptosis, while the myocardial histopathological changes were mitigated after WETA treatment (*p* < 0.01) (Figure 5A,B). Masson’s trichrome staining (Figure 5C,D) indicated extensive myocardial fibrosis in the ISO group (*p* < 0.01), and WETA significantly reduced the myocardial fibrosis area (*p* < 0.01) (Figure 5C,D). These results illustrated that WETA could attenuate inflammatory infiltration and reduce myocardial fibrosis in ISO-induced rats.

### 2.6. Effects of WETA on Inhibiting Inflammatory Response

**Figure 6:**
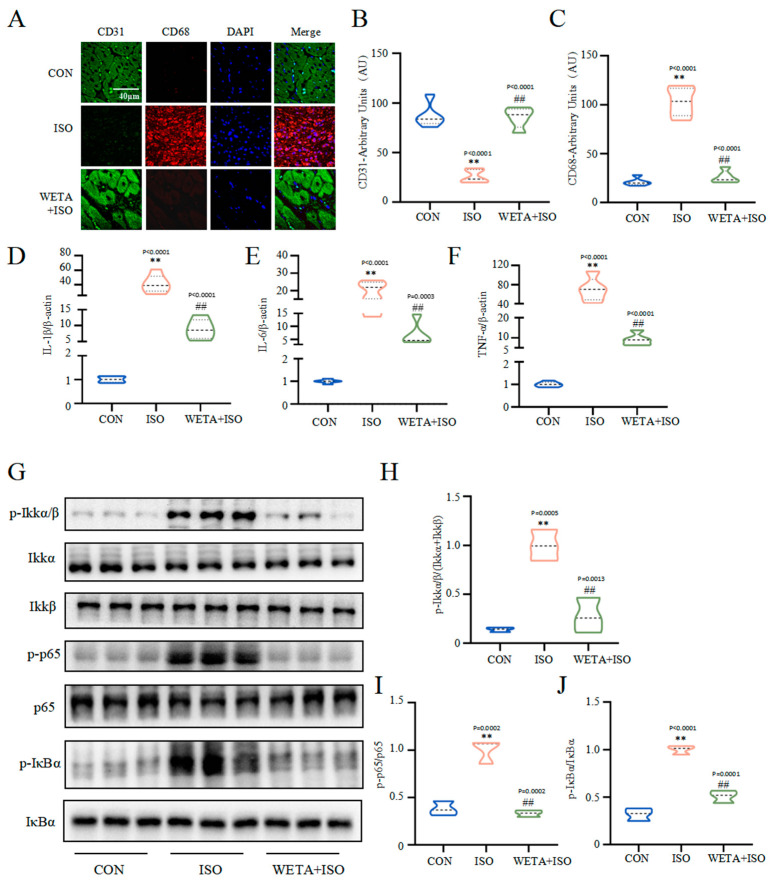
WETA attenuated the inflammatory response in heart tissues of ISO-induced rats. (**A**) Immunofluorescence staining images of CD68 (red), CD31 (green), and nuclei (blue) (scale bar = 40 μm). Mean fluorescence intensity of CD68 (**B**) and CD31 (**C**); mRNA levels of IL-1β (**D**), IL-6 (**E**), and TNF-α (**F**). (**G**) Western blot results of the levels of p-Ikkα/β (**H**), p-p65 (**I**), and p-IκBα (**J**). Values are mean ± SD (n = 6), ** *p* < 0.01 vs. CON group; ^##^ *p* < 0.01 vs. ISO group, using one-way ANOVA.

As shown in Figure 6, the immunofluorescence results showed that the expression of macrophage marker CD68 was increased (*p* < 0.01) and endothelial cell marker CD31 was decreased (*p* < 0.01) in the ISO group, which were reversed by WETA treatment (*p* < 0.01). With the activation of macrophages, the mRNA level of pro-inflammatory factors, including IL-1β, IL-6, and TNF-α were elevated (*p* < 0.01) by ISO. After WETA treatment, the expressions of pro-inflammatory factors were significantly decreased (*p* < 0.01). Moreover, ISO promoted the phosphorylation of Ikkα/β, p-65, and IκBα (*p* < 0.01), and the activation was suppressed by WETA (*p* < 0.01). These results supported that WETA could inhibit ISO-induced cardiac inflammation by inhibiting the release of inflammatory factors and suppressing the activation of the NF-κB signaling pathway.

### 2.7. Effects of WETA on Improving Myocardial Damage

**Figure 7:**
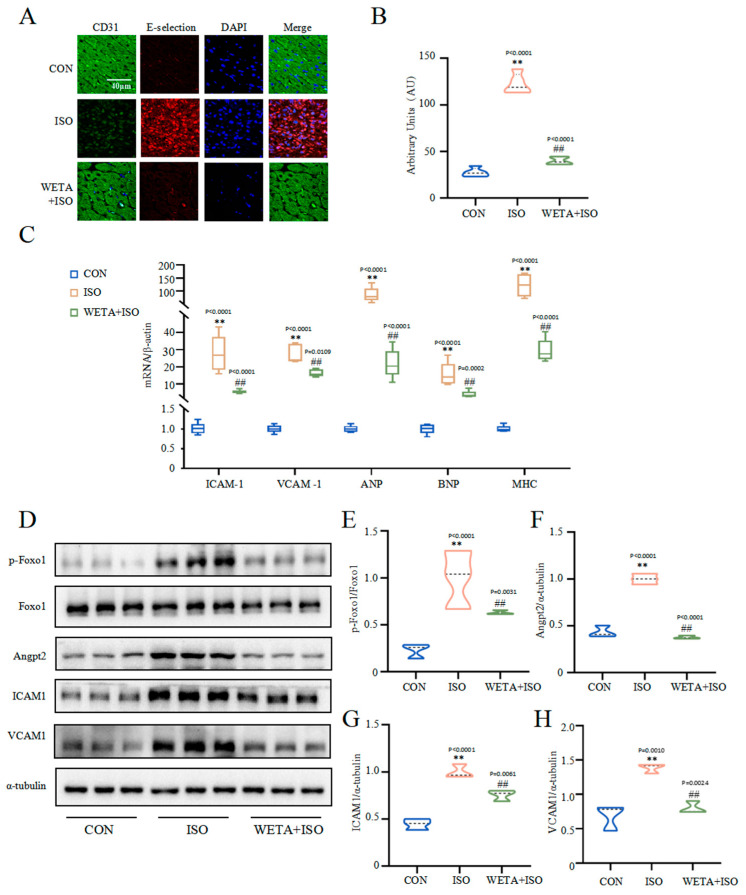
WETA attenuates cardiac myocardial damage. (**A**) Immunofluorescence staining images of E-selection (red), CD31 (green), and nuclei (blue) (scale bar = 40 μm), (**B**) mean fluorescence intensity of E-selection, (**C**) expression of ANP, BNP, MHC, ICAM1, and VCAM1. (**D**) Western blot results of levels of p-Foxo1 (**E**), Angpt2 (**F**), ICAM1 (**G**) and VCAM1 (**H**). Values are mean ± SD (n = 6), ** *p* < 0.01 vs. CON group; ^##^ *p* < 0.01 vs. ISO group, using one-way ANOVA.

The immunofluorescence staining (Figure 7A,B) showed that the adhesion factor E-selectin in the myocardial tissue of the ISO group was increased (*p* < 0.01), accompanied by increased mRNA levels of VCAM1 and ICAM1 (*p* < 0.01) (Figure 7C), suggesting myocardial cell damage. However, WETA administration mitigated the expressions of E-selection, VCAM1, and ICAM1 (*p* < 0.01). Furthermore, ISO significantly caused up-regulation of cardiac hypertrophy markers, including ANP, JNP, and MHC, which were suppressed by WETA administration (*p* < 0.01) (Figure 7C). The Western blot results showed that p-Foxo1 and its downstream factor Angpt2 were activated by ISO treatment (*p* < 0.01) (Figure 7E,F), while WETA could inhibit the increase in p-Foxo1 and Angpt2 (*p* < 0.01) (Figure 7E,F). These results demonstrated that WETA alleviated myocardial dysfunction by suppressing the expression of cardiac hypertrophy markers and adhesion factors and suppressing the expression of Angpt2.

## 3. Discussion

HF is the most common complication following myocardial infarction and is directly relevant to the development of ventricular remodeling [1]. Currently, the commonly prescribed medications for HF management are angiotensin-converting enzyme inhibitors (ACEIs), β-adrenergic blockers, and diuretics [23]; however, prolonged administration of these drugs cause serious side effects, like hypotension and bradycardia [24]. Therefore, the development of novel drugs for the treatment of HF is critical. Traditional Chinese medicine has the characteristics of good efficacy, minimal effects, and effects on many targets. *Aconitum carmichaelii* Debx., one of plants used in traditional Chinese medicine, has been used to treat cardiovascular disease for centuries in China.

Structural, functional, and geometric alterations in the myocardium ultimately lead to cardiac dysfunction [1]. The systolic function of the LV plays an invaluable role in the diagnosis and evaluation of cardiac disease [25]. LVEF provided by echocardiography is one of the most traditional clinical metrics of HF with reduced EF [26], and is usually coupled with a significant increase in ventricular dilatation that is assessed as the diameters of the ventricles becomes larger during end-systole and end-diastole or as LV volumes, compared with health patients [27]. These alterations were also observed in a rat model of HF [28]. In the present study, a significant reduction in ES and FS, and an increase in LVIDs, LVIDd, LVESV, and LVEDV were detected in an ISO-induced rat model of HF, indicating the dysfunction of the LV and myocardial injury. Additionally, these parameters were significantly reversed by WETA treatment. Strain echocardiography is another strong predictor to assess myocardial function [25]. Based on the strain analysis, the rats in the ISO group exhibited reduced RRS and increased RLS, reflecting impaired LV systolic function and myocardial function, while WETA improved the changes in RRS and RLS. These findings demonstrate the protective effect of WETA on the ISO-stimulated myocardium.

Clinical risk stratification for heart disease and cardiac injury is usually performed using cardiac injury markers [29]. The most important biomarkers for the diagnosis of myocardial necrosis in the clinic, such as cTnT, cTnI, and CK-MB, are released from damaged cardiomyocytes into the blood [30]. Similarly, structural impairment of the cardiomyocyte membranes and increased myocardial injury biomarkers were observed in rats with HF [31]. Notably, the levels of myocardial enzymes in the WETA group were significantly alleviated, which may be connected with the protective effect of WETA on cardiomyocyte membranes. Moreover, HF also manifests as a hypertrophic heart, larger infarct sizes, infiltration of inflammatory cells, and collagen deposition [32,33]. These changes in the cardiac tissues in ISO-induced rats were significantly ameliorated by WETA treatment. Altogether, these results reveal a protective role of WETA on heart tissues.

Inflammation is a key process in the pathogenesis of HF [34]. Previous studies showed that infiltration of immune cells in infarcted myocardium drives inflammation and further promotes pro-inflammatory cell recruitment [35,36] and induces myocardial cell death and myocardial damage, therefore aggravating cardiac remodeling and accelerating the development of HF [35,37]. In line with this knowledge, we found a large number of macrophages aggregated in heart tissues, and the expressions of IL-1β, IL-6, and TNF-α were increased in the ISO group, whereas WETA treatment attenuated macrophage accumulation and the expression of these pro-inflammatory mediators. In addition, NF-κB activation in the myocardium enhances the infiltration of macrophages [38,39] and mediates the release of pro-inflammatory factors and adhesion factors that exacerbate cardiac remodeling in both patients and rats with HF [40]. In the current study, the phosphorylation levels of Ikkα/β, p65, and IκBα were significantly increased in the ISO group, which were suppressed by WETA. These results suggest that WETA may suppress ISO-induced cardiac inflammation by inhibiting the NF-κB signaling pathway.

Abnormal myocardial vessel remodeling is the foundation of the occurrence and development of HF [40]. The sustained pathological overload of cardiovascular disease stimulates cardiomyocyte hypertrophy that leads to progressive ventricular dilatation and further deteriorates cardiac function into HF [41]. Cardiac hypertrophic markers, such as ANP, BNP, and MHC, were significantly increased in heart tissues of ISO-induced rats, and WETA application significantly attenuated these abnormal expression patterns, thereby preventing cardiac remodeling. Moreover, the hypertrophic myocardium also secretes vascular growth factors, which stimulate vascular growth to cope with abnormal myocardial dilatation [42]. Angpt2, a recognized vascular instability factor and a facilitator of vascular inflammation, is highly expressed in the myocardial injury regions and promotes abnormal vascular remodeling [15,43], which participates in vascular leakage, increased expression of adhesion molecules, and augmented inflammatory cell infiltration in the infarcted cardiac tissue [15]. Here, we found that the expression of Angpt2 and the phosphorylation of its upstream factor FOXO1 were increased in the ISO group, whereas WETA intervention counteracted these phenomena. In addition, the abnormal vessels promote TNF-α expression, thereby enhancing the expression of ICAM1, VCAM1, and E-selection, which exacerbates vascular adhesion and aggravates vascular damage [44,45]. Indeed, these adhesion factors were highly expressed in heart tissues of ISO-induced rats, while these genes were markedly reduced in the WETA group. Taken together, WETA could prevent deterioration to HF via inhibiting cardiac hypertrophy and abnormal vascular remodeling.

There are several limitations concerning this study that should be noted. Firstly, an explicit demonstration of how WETA inhibits inflammatory responses and alleviates vascular injury in cardiac tissues via the NF-κB and FOXO1/Angpt2 signaling pathways needs to be further explored. Secondly, the current experiment is mainly based on the preventive effect of WETA; the exploration of the roles and mechanisms of WETA in alleviating the consequences of HF is a topic for further study.

## 4. Methods

### 4.1. The Extraction Process of Aconitum carmichaelii Debx.

The toasted *Aconitum carmichaelii* Debx., purchased from Sichuan Jiangyou Zhongba Aconite Technology Development Co., Ltd. (Jiangyou, China), was weighted and decocted with 10 equivalent masses of double-distilled water (1:10, *w/v*) for 5 h, and then filtered. Subsequently, 8 equivalent volumes of double-distilled water were added, and cooked for another 3 h. After filtration, the two fluids were mixed and concentrated.

### 4.2. Qualitative Analysis of WETA

A total of 100 mg of WETA was weighed and added to 1 mL of methanol; after being shaken and mixed, the supernatant was filtered to obtain the sample. The compounds in WETA were determined using Vanquish Ultra Performance Liquid Chromatography coupled with a Q Exactive Quadrupole High resolution mass spectrometer with an electrostatic field orbit trap (Thermo Fisher Scientific, Waltham, MA, USA).

The mobile phases are composed of formic acid (0.1%) with aqueous (A) and formic acid (0.1%) with acetonitrile (B). A Thermo Scientific Accucore^TM^ C18 column (3 mm × 100 mm, 2.6 μm) was used with a flow rate of 0.5 mL/min, column temperature of 25 °C, and sample injection volume of 8 μL. The gradient elution program was set to 0–20 min, 2–50% B; 20–20 min, 50–90% B; 32–40 min, 95% B. The sample was detected in positive ion mode using an electrospray ion source (ESI). The following values were chosen as the optimal ion source parameters for mass spectrometry analysis in positive ionization mode: ion spray voltage, 3500 V; ion source temperature, 380 °C; sheath gas, 35 arb; auxiliary gas, 10 arb; ion transport tube temperature, 320 °C. A mass spectrum range of 100–1500 **m*/*z** was found to be optimal for compound separation. Data were analyzed by X Calibur 3.0 software (Thermo Fisher Scientific, Waltham, MA, USA) and Compound Discoverer 2.0 software coupled to mvCloud© and mzVault© databases.

### 4.3. Experimental Animals and Treatment

The experiments were approved by the ethics committee of Chengdu University of Traditional Chinese Medicine (No. 2021-48). Eighteen male Wistar rats (200–220 g) were obtained from SPF (Beijing, China) Biotechnology Co., LTD (Beijing, China, No. SCXK 2019-0010), and kept under control conditions (23 ± 2 °C) in an animal house with a 12 h dark/light cycle, and provided ad libitum water and adequate food. All the animals were randomly divided into three groups: the Control group (CON), the Isoproterenol group (ISO), and the WETA group (WETA + ISO). The rats in WETA group were intragastrically (i.g) administrated with WETA (10 mL/kg), and the CON and ISO were given the same volume of sterile double-distilled water for 14 continuous days. Then, the rats in ISO and WETA were intraperitoneally injected (i.p) with ISO (SIGMA, Livonia, MI, USA) (5 mg/kg/day) for 7 days, while the CON group was administrated saline. The experimental design is shown in Figure 1A.

### 4.4. Echocardiographic Analysis

Rats were anesthetized by inhalation of 3% isoflurane (RWD Life Science Co., Ltd., Shenzhen, China), and a Vevo 3100 system (FUJIFILM VisualSonics, Toronto, ON, Canada) was utilized to detect the function of LV. The LV ejection fraction (EF), LV fractional shortening (FS), LV end-diastolic internal dimension (LVIDd), LV end-systolic internal dimension (LVIDs), LV end-diastolic volume (LVEDV), and LV end-systolic volume (LVESV) were collected from the M-mode images.

### 4.5. Speckle-Tracking Echocardiographic

The B-Mode images of the long axis were acquired from a Vevo 3100 system (FUJIFILM VisualSonics, Toronto, ON, Canada), and analyzed using the Vevo Strain Software (Fujifilm VisualSonics, Toronto, ON, Canada) to obtain regional radial strain (RRS) and regional longitudinal strain (RLS). The long axis of the LV was automatically segmented into six parts by the strain software.

### 4.6. Collection and Detection of Cardiac Tissue

After rats were euthanized, the blood samples were collected to harvest sera, which were stored at −80 °C. Then, the cardiac tissues were immediately removed and washed with pre-cooled saline after the connective tissues were cut out. Subsequently, the cardiac tissues were weighed and photographed. Additionally, the cardiac weight index (CWI) was calculated according to the following formula: CWI (g/g) = heart tissue weight (g)/body weight (g) × 100.

### 4.7. TTC Staining and Measurement of the Infarction Area

The heart tissues were frozen at −20 °C for 20 min and cut into 4 mm thick slices and incubated at 37 °C in 2% TTC (Solarbio, Beijing, China) staining solution for 30 min. The result of the staining was then observed and the heart sections were fixed in 4% paraformaldehyde. After that, the infarction areas were photographed and analyzed using image J software.

### 4.8. Determination of Serum Biomarkers

The cardiac function biomarkers creatine kinase MB (CK-MB), cardiac troponin T (cTnT), and cardiac troponin I (cTnI) were detected by ELISA kits (Elabscience Biotechnology Co. Ltd., Wuhan, China) according to the manufacturer’s protocols.

### 4.9. H&E Staining and Masson’s Trichrome Staining

The heart tissues were fixed in 4% paraformaldehyde, embedded in paraffin, and cut into 5 μm thick slices. The slices were stained using a hematoxylin and eosin (H&E) staining kit (Beyotime, Haimen, China) according to the manufacturer’s instructions. The cross-sections of the cardiac tissues were stained with Masson’s trichrome to detect the fibrosis degree of the heart. Six microscopic fields of view were randomly selected and photographed using a digital camera (Nikon, Tokyo, Japan) linked to a microscope (Nikon, Tokyo, Japan) to analyze the pathological changes and degree of fibrosis of the heart. Collagen volume fraction (CVF) was analyzed by Image J. CVF = collagen area of myocardial interstitium/total field area. The extent of inflammatory infiltration was defined by the number of cells per square millimeter [46].

### 4.10. qRT-PCR Analysis

The cardiac tissues were homogenized, and the total RNA was extracted using TRIzol reagent (Invitrogen, Waltham, MA, USA) and reversed transcribed with a reverse transcription kit (ThermoFisher Scientific, Waltham, MA, USA). An ABI StepOnePlus PCR system (ThermoFisher Scientific, Waltham, MA, USA) was used to detect the expression levels of related genes. The gene specific primers used in this study are listed in Table 2. The relative mRNA expressions of genes were calculated by the 2^−△△CT^ method as previously described [47].

### 4.11. Immunofluorescence Analysis

After dewaxing and rehydration, the 5 μm thick sections were boiled in 0.1 M sodium citrate buffer (pH = 6.0) for 30 min for antigen repair. After washing three times with PBS, the slices were blocked with 10% BSA for 1h, and subsequently incubated with primary antibodies at 4 °C overnight. After that, the slices were stained in fluorescent secondary antibody (ThermoFisher Scientific, Waltham, MA, USA) for 1 h in darkness. After rinsing three times with PBS, the nucleus was counterstain with DAPI for 20 min. The slides were examined with a confocal fluorescence microscope (Olympus, Tokyo, Japan). The information for the antibodies is shown in Table 3.

### 4.12. Western Blot Analysis

In brief, cardiac tissues were homogenized and lysed in RIPA buffer (Beyotime, Shanghai, China) containing a protease cocktail and PMSF. The total protein concentration was detected using a BCA kit (Thermo Fisher Scientific, Waltham, MA, USA). The samples were separated on 8% SDS-polyacrylamide gels and transferred onto PVDF membranes, then blocked with 5% skim milk for 1 h. Thereafter, the membranes were incubated in the primary antibodies at 4 °C overnight. After secondary antibody incubation for 2 h, the proteins were visualized in an imaging system (Tanon, Hangzhou, China) using ECL reagents (Thermo Fisher Scientific, Waltham, MA, USA) to detect the immunoblot signals, which were analyzed with Image J software. The information for the antibodies are shown in Table 2.

### 4.13. Statistical Analysis

All data were presented as mean ± SD and analyzed with GraphPad Prism 8.2.1 software (San Diego, FL, USA). One-way ANOVA was used to examine the intergroup comparisons [48], the normal distribution of data was analyzed using the D’Agostino–Pearson test, and multiple comparisons were then performed using the Dunnett t-test. Additionally, when *p* values were lower than 0.05, the results were deemed statistically significant.

## 5. Conclusions

In summary, our results indicate that the WETA could improve ISO-induced HF by improving heart function, inhibiting inflammatory responses, and alleviating vascular injury in cardiac tissues via the NF-κB and FOXO1/Angpt2 signaling pathways. Thus, Aconitum carmichaelii Debx. may be a promising candidate for the treatment of heart failure.

## Figures and Tables

**Table 1:** The identification of bioactive compounds in WETA by full MS/dd-MS^2^ [22].

Peak No.	t_R_ (min)	Compounds	Molecular Formula	Calculated MS (*m*/*z*)	Observed (*m*/*z*)	Error (ppm)
1	6.418	Salsolinol	C_10_H_13_NO_2_	179.09463	179.09502	2.18
2	7.937	Karakoline	C_22_H_35_NO_4_	378.2635	377.25716	1.46
3	10.377	Isotalatizidine	C_23_H_37_NO_5_	408.2742	407.26772	1.34
4	10.812	Napelline	C_22_H_33_NO_3_	360.2525	359.24672	1.89
5	11.98	Neoline/Bullatine B	C_24_H_39_NO_6_	438.2862	437.27834	1.37
6	12.103	Neoline	C_24_H_39_NO_6_	437.27774	437.27834	1.37
7	12.177	Lycoctonine	C_25_H_41_NO_7_	468.2955	467.28935	2.25
8	12.289	Fuziline (15-*α*-Hydroxyneoline)	C_24_H_39_NO_7_	453.27265	454.2793	1.5
9	12.969	Talatisamine	C_24_H_39_NO_5_	421.28282	421.28365	1.96
10	16.770	Benzoylmesaconine	C_31_H_43_NO_10_	589.47383	589.28905	0.6
11	17.980	Benzoylaconine	C_32_H_45_NO_10_	603.30435	603.30528	1.55
12	18.566	Hypaconitine	C_33_H_45_NO_10_	615.30435	615.30461	0.43
13	18.590	Benzoylhypaconine	C_31_H_43_NO_9_	573.29378	573.29418	0.69
14	19.410	Aconitine	C_34_ H_47_ NO_11_	645.31491	645.31664	2.67

**Table 2:** Sequences of primers used in the gene expression analysis.

Gene	Primer Sequence (5′ to 3′)
TNF-α	F: TACTCCCAGGTTCTCTTCAAGG
	R: GGAGGCTGACTTTCTCCTGGTA
IL-6	F: GAGTTGTGCAATGGCAATTC
	R: ACTCCAGAAGACCAGAGCAG
IL-1β	F: CACCTCTCAAGCAGAGCACAG
	R: GGGTTCCATGGTGAAGTCAAC
ANP	F: CTGCTAGACCACCTGGAGGA
	R: AAGCTGTTGCAGCCTAGTCC
BNP	F: GATCCAGGAGAGACTTCGAAA
	R: CGGTCTATCTTCTGCCCAA
MHC	F: GAGGAGAGGGCGGACATT
	R: ACTCTTCATTCAGGCCCTTG
ICAM-1	F: AGATCATACGGGTTTGGGCTTC
	R: TATGACTCGTGAAAGAAATCAGCTC
VCAM-1	F: TTTGCAAGAAAAGCCAACATGAAAG
	R: TCTCCAACAGTTCAGACGTTAGC
β-actin	F: GAAGTGTGACGTTGACATCCG
	R: TGCTGATCCACATCTGCTGGA

**Table 3:** Antibody information used in the Western blot and immunofluorescence analysis.

Antibodies	Source	Production Company	Catalog Numbers
anti-phospho-IKKα/β (Ser176/180)	Rabbit	Cell Signaling Technology	#2697
anti-IKKα	Mouse	Cell Signaling Technology	#11930
anti-IKKβ	Rabbit	Cell Signaling Technology	#8943
anti-phospho-IκBα (Ser32)	Rabbit	Cell Signaling Technology	#2859
anti-IκBα	Mouse	Cell Signaling Technology	#4814
anti-phospho-p65 (Ser536)	Rabbit	Cell Signaling Technology	#3033
anti-p65	Rabbit	Cell Signaling Technology	#8242
anti-p-Foxo1	Rabbit	Cell Signaling Technology	#2599
anti-Foxo1	Rabbit	Cell Signaling Technology	#2880
anti-VCAM1	Rabbit	ABclonal	A11236
anti-ICAM1	Rabbit	ABclonal	A5597
anti-CD68	Rabbit	ABclonal	A6554
anti-E-selectin	Rabbit	proteintech	20894-1-AP
anti-CD31	Mouse	proteintech	66065-2-Ig
anti-Angpt2	Rabbit	affinity	#DF6137
α-Tubulin	Mouse	ABclonal	AC012
Anti-rabbit IgG, HRP-linked Antibody	Goat	Cell Signaling Technology	#7074
Anti-mouse IgG, HRP-linked Antibody	Horse	Cell Signaling Technology	#7076
Alexa Flour 488 goat anti-mouse IgG	Goat	Thermo Fisher Scientific	A11029
Alexa Flour 594 goat anti-Rabbit IgG	Goat	Thermo Fisher Scientific	A11037

## Data Availability

The data used to support the findings of this study are available from the corresponding authors upon request.
